# Towards a universal understanding of post COVID-19 condition

**DOI:** 10.2471/BLT.21.286249

**Published:** 2021-10-05

**Authors:** Janet V Diaz, Margaret Herridge, Silvia Bertagnolio, Hannah E Davis, Tarun Dua, Charu Kaushic, John C Marshall, Maria del Rosario Pérez, Nathalie Strub-Wourgaft, Joan B Soriano

**Affiliations:** a Clinical Management Team, World Health Organization, Avenue Appia 20, 1211 Geneva 27, Switzerland.; b Department of Medicine, Interdepartmental Division of Critical Care, University of Toronto, Toronto, Canada.; c Department of Control, Surveillance and Prevention of Antimicrobial Resistance, World Health Organization, Geneva, Switzerland.; d Patient-Led Research Collaborative, United States of America.; e Brain Health Unit, World Health Organization, Geneva, Switzerland.; f CIHR Institute of Infection and Immunity, McMaster University, Hamilton, Canada.; g Departments of Surgery and Critical Care Medicine, St Michael's Hospital, Toronto, Canada.; h Drugs for Neglected Diseases initiative, Geneva, Switzerland.

As of November 2021, the global number of cumulative coronavirus disease 2019 (COVID-19) positive cases had surpassed 247 million, with a death toll of 5 million.^1^ While many countries have started vaccination programmes and more than 5 billion doses of available vaccines have been administered since December 2020,^2^ vaccine distribution has been inequitable. All parties are aiming for a wider and faster distribution worldwide; however, controlling COVID-19 will require a multifaceted, global collaboration of vaccination programmes and implementation of public health and social measures at individual and community levels.^3^

Emerging evidence warns of the concerning long-term effects following infection with severe acute respiratory syndrome coronavirus 2 (SARS-CoV-2).^4^ According to the limited amount of available published clinical series, approximately 80% of patients with COVID-19 have symptoms that persist beyond 2 weeks.^5^^,^^6^ Multiple symptoms are being described by patients in an increasing number of surveys.^5^^–^^7^ Symptoms range from cough and shortness of breath to fatigue, exertional malaise, chest pain, joint pain, headache, depression or other mood disorders, cognitive dysfunction and insomnia. Multiorgan system involvement might exist, including but not limited to neurological, mental, cardiovascular, musculoskeletal, reproductive and renal systems. Patients who experienced hospitalization during the acute episode of COVID-19 as well as patients who had a milder disease not requiring hospitalization have reported such symptoms.

In December 2020, two independent virtual forums were conducted on this topic by the International Severe Acute Respiratory and emerging Infection Consortium and Global Research Collaboration for Infectious Disease Preparedness,^8^ and the National Institutes of Health/National Institute of Allergy and Infectious Diseases.^9^

**Fig. 1:**
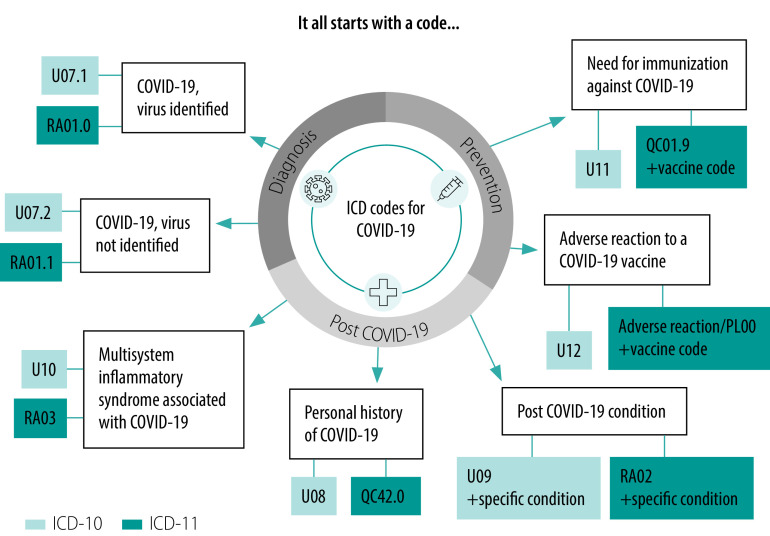
Emergency use ICD codes for COVID-19 disease outbreak

COVID-19: Coronavirus disease 2019; ICD: International Classification of Diseases.

Source: World Health Organization.^10^

In September 2020, the World Health Organization (WHO) convened a consultation with Member States on the International Classification of Diseases ICD-10 and ICD-11 codes (Fig. 1),^10^ and proposed the use of the term post COVID-19 condition to diagnose and code patients with this condition. This terminology does not attribute causality and does not refer to any duration, unlike other terms such as chronic COVID-19 syndrome; late sequelae of COVID-19; long COVID; long haul COVID; long-term COVID-19; post COVID syndrome; post-acute COVID-19; post-acute sequelae of SARS-CoV-2 infection and others. Adding post COVID-19 condition to the classification system, along with the strong advocacy conducted by patient advocacy groups such as LongCovidSOS have increased the recognition of this condition. 

On 9 February 2021, WHO in collaboration with the International Severe Acute Respiratory and emerging Infection Consortium and the Global Research Collaboration for Infectious Disease Preparedness, the National Institutes of Health and the National Institute of Allergy and Infectious Diseases, LongCovidSOS and others, convened an open global webinar in a series entitled *Expanding our understanding of post COVID-19 condition*. The webinar included patients, clinical and basic scientists, the media and diverse stakeholders from around the world. Participants shared their experience, presented the latest research results and identified critical research gaps to investigate the frequency, burden and sequelae following acute SARS-CoV-2 infection.^11^ The virtual forums held during the webinar were designed to identify knowledge gaps, promote improved collective learning and mobilize resources for the prevention, treatment and care of post COVID-19 condition.

This first WHO webinar aimed to address the priority of developing a working clinical case definition and to harmonize treatment, surveillance and awareness strategies across the clinical, research, public health and patient advocacy communities.^12^ Doing so remains a challenging task, as the natural history and spectrum of manifestations related to SARS-CoV-2 infection and COVID-19 are not yet well understood. This webinar held simultaneous working group sessions that attempted to develop a working clinical case definition by consensus of the participating audience. The foundational work was initiated, but the development of a case definition is an ongoing process. WHO will request further inputs using a methodologically rigorous approach through a Delphi process.^13^ More evidence is needed to determine the relationship between the time course from exposure to infection and the duration and severity of the acute episode of COVID-19 on the one hand, and the recovery from or persistence of the post COVID-19 condition on the other. In addition, the effects of reinfection, vaccination and symptoms that are temporally and causally related to COVID-19 need to be defined. Major questions remain, among others: What are the risk factors associated with post COVID-19 condition? Could early treatment prevent post COVID-19 condition? What are the pathogenic mechanisms for symptoms depending on the severity of the infection? What causes the multisystemic and neurological symptoms seen in the post COVID-19 condition?

Fewer than 1% (45/5000) of ongoing COVID-19 research studies are focused on studying post COVID-19 condition ‒ or its associated terminologies.^14^ Most studies are based on patients from hospital series. For this reason, new research from primary care and community-based settings is essential to incorporate the experience of those patients who were less likely to be hospitalized, including younger patients, and those who had either a mild infection with fewer symptoms or were undiagnosed.^15^ Including these cases is important, as some evidence seems to suggest that the initial severity of the acute disease may not be associated with the incidence or severity of post COVID-19 condition.^16^ Furthermore, low antibody titres may be correlated with persistent symptoms and patients with low or no antibodies should be included in research.^17^ High heterogeneity in the manifestations and severity of infection with SARS-CoV-2 (from asymptomatic to severe COVID-19) exists, and this heterogeneity is observed as well in many patients with post COVID-19 condition.^18^ Yet, the pain and suffering of many of those living with post COVID-19 condition and their caretakers is consistent.^19^ We need to understand this heterogeneity; the interconnectedness of multiple potential pathophysiologic mechanisms including sustained alterations in innate and adaptive immunity; effects on the central and peripheral nervous system; abnormalities of thrombosis and endothelial function; and other biological processes underlying prolonged organ-specific symptoms. This list is not exhaustive and some of these mechanisms are likely to be interdependent. Importantly, any early phenotypes must leave room for the expansive, heterogenetic nature of this condition and should not be reductive until we understand more about this condition.

Leaving no one behind^20^ is fundamental for those who have had COVID-19 and are now living with its consequences, in some cases in solitude and with psychosocial mental distress.^21^ New investigations must include a focus on children, women, older adults, diverse ethnic origins and socioeconomically disadvantaged groups and marginalized populations including incarcerated individuals, migrants, refugees and all those suffering stigma and discrimination. COVID-19 should not be an aggravating factor to their status.

Learning the lessons from past (such as from sequelae post 1918 influenza)^22^ or more recent outbreaks (post Zika, Chikungunya, SARS or Middle East respiratory syndrome),^23^ and from methodological approaches used to define the post-intensive care syndrome or systemic exercise intolerance syndrome, we are able to advance this work more quickly. Relevant post-viral disease research done on systemic exercise intolerance syndrome in the areas of neuroimmunology, metabolic profiling, impaired endothelial function, intracranial hypertension, altered B- and T-cells, metabolomics and proteomics, autoimmunity, hypermobility and others were highlighted during the first WHO webinar as areas to continue post-viral disease investigation.

Additionally, raising awareness on post COVID-19 condition and providing resources for patients and those in decision-making roles is needed.^24^^,^^25^ Evidence from multiple sources will need to be synthesized to inform guideline development and to implement and evaluate coordinated, multidisciplinary models of care that will fit the needs of these patients.^26^

Finally, this work must be multicentre and multidisciplinary, and involve international cooperation. Collecting standardized and harmonized data on post COVID-19 condition will be paramount, as available published studies are small, heterogeneous in nature, too short in their follow-up, and with narrow focus on a few organ systems assessed. To support systematic collection of comparable data across the world, WHO has developed a Global Clinical Platform and standardized case report forms for acute COVID-19 and post COVID-19 condition that are available for all Member States and interested parties.^27^ Data should be synthesized to extend the available collective experience with this condition and its insights, and should be meta-analysed to enlarge the available experience from a few hundred person-years’ experience to many more orders of magnitude. Data sharing should be undertaken in the broadest sense, to include experience in all affected patients and in subgroups by demographic and clinical variables. Technologies analysing large data sets and tools of artificial intelligence applied directly to text of electronic health records are already available to be used.^28^ Follow-up from existing trials that randomized patients less than one year ago could be of enormous value, with little investment required to support data collection. The recent National Institutes for Health announcement of specific funds and setting up a biospecimen bank will steer research and researchers on this topic.^29^ Multinational data sharing should be promoted to ensure broad demographics, variable case mix with diverse genetics and the effects of distinct health-care systems and access to treatments. Making sense of heterogeneous data sources and their appropriate interpretation are both a challenge and part of the final solution to better understand post-COVID-19 condition.^30^

WHO is committed to engaging with academic and research communities, advocacy groups, patients and their representatives, and health research funders to ensure success with research and care on post COVID-19 condition.^31^
